# Hear, See, Do (Nothing)? An Integrative Framework of Co-Workers’ Reactions to Interpersonal Workplace Mistreatment

**DOI:** 10.3390/bs15060764

**Published:** 2025-06-02

**Authors:** Caroline Veronique Marijke Bastiaensen, Elfi Baillien, Lieven Brebels

**Affiliations:** Work and Organisation Studies, KU Leuven, 1000 Brussels, Belgium; elfi.baillien@kuleuven.be (E.B.); lieven.brebels@kuleuven.be (L.B.)

**Keywords:** interpersonal workplace mistreatment (IWM), co-worker reactions, dual processing accounts, integrative framework, affective events theory, attribution theory

## Abstract

Interpersonal workplace mistreatment (IWM) is a pervasive issue with varied co-worker reactions. Despite significant growth in IWM research, understanding co-workers’ responses remains challenging due to scattered research streams and a focus on isolated components. Therefore, this integrative review synthesizes the existing literature to examine when and how co-workers respond to IWM. We identify key factors influencing both the intention and actual behavior of co-workers when initially witnessing IWM. These factors are categorized into eight overarching individual (perceived responsibility, emotion and affect, personal characteristics) and contextual themes (social relationships, risks and costs considerations, workplace characteristics, target characteristics, characteristics of the perpetrator and the act of IWM). By integrating these factors into a comprehensive framework drawing on dual processing accounts, we highlight the complex interplay between cognitive and affective processes in shaping co-worker responses. Understanding these dynamics is crucial for designing effective intervention programs that encourage co-workers to counteract IWM. Our findings offer valuable theoretical insights and practical implications for fostering a more supportive and just workplace environment.

## 1. Introduction

In recent years, the research field of interpersonal workplace mistreatment (IWM) has seen significant growth. IWM encompasses different concepts and literature streams, and it is thus considered an umbrella term for negative social acts in the work context, oriented from at least one employee towards another, that have negative consequences for the target(s) of the act (Cortina & Magley, 2003; Dhanani & LaPalme, 2019) (for further elaboration, see pages 2–3). Besides looking at antecedents and consequences, researchers have increasingly focused on the various co-workers’ responses to witnessing such acts (Reich et al., 2021). This interest stems from the awareness of IWM as a social phenomenon including several social actors beyond ‘just’ actors and targets (Dhanani & LaPalme, 2019; Ng et al., 2020). Studies on co-workers have revealed that these third parties can significantly contribute to the escalation of IWM by either remaining passive observers or by actively taking part in the IWM (Zhou et al., 2021). However, co-workers can also play a significant role in de-escalating the mistreatment situation (Kuntz & Searle, 2023). This has sparked scientific interest in understanding and potentially altering co-workers’ behavioral responses, aiming to craft co-worker interventions that can effectively mitigate IWM in the workplace.

Despite the increased attention for co-worker responses, we see two challenges that hinder a comprehensive understanding of these responses, and, consequently, the development of effective interventions. Firstly, research on co-worker responses to IWM is scattered across a wide range of separate research streams (e.g., on incivility, bullying, workplace aggression, or social undermining) (Lim & Cortina, 2005) with notably little cross-fertilization (Dhanani & Bogart, 2025; Hershcovis, 2011). This fragmentation hampers theoretical integration, limits cumulative knowledge development, and makes it difficult to identify overarching mechanisms—such as co-worker decision-making processes—that may operate similarly across different forms of mistreatment. Secondly, empirical studies to date have predominantly examined isolated components or specific variables of interest (e.g., the role of empathy, moral identity, or attributions of responsibility) without truly unraveling the co-workers’ decision-making processes when confronted with IWM. Consequently, the majority of the existing knowledge focuses on the types of co-worker behavioral reactions and their effects on the IWM situation and/or the target (Pouwelse et al., 2018). However, this approach has failed to acknowledge the internal processes within the co-worker leading to these behaviors. Establishing the insights necessary for developing successful intervention programs targeting co-workers’ responses requires a comprehensive understanding of these internal processes. If we seek to influence co-workers’ behavioral reactions to witnessing IWM using interventions, we must first gain insights into how and why the decisions to respond this way are made.

To address these significant gaps, this paper aims to integrate the disparate elements influencing co-workers’ behavioral responses towards their first confrontation with IWM as identified in the silos. By drawing from empirical findings and theoretical elaborations across the research streams, we aim to (a) present a state-of-the-art summary of influencing factors and (b) create an integrative framework encompassing a more programmatic perspective on co-workers’ responses to a first confrontation with witnessing IWM (Cronin et al., 2021). We explicitly apply a decision-making lens to illustrate the pathways in co-workers’ decision-making processes, which yield several important contributions to the field. Understanding not only what influences decisions but also how these decisions are made is essential for designing programs or interventions that can effectively alter co-workers’ responses (E. U. Weber et al., 2004). First, this framework serves as a guide for further empirical research, validating the several components of our integrative framework. This will help establish a strong knowledge base regarding factors that contribute to the co-workers’ decision-making processes with their initial response to witnessing IWM. Second, the framework can inspire more finely tuned interventions aimed at preventing and de-escalating IWM situations.

## 2. A Much-Needed Integrated Co-Workers’ Perspective on IWM

IWM is a flourishing research area. Notably, in the scientific literature, acts of interpersonal workplace mistreatment are often studied as specific constructs, such as deviant workplace behavior (Robinson & Bennett, 1995), workplace aggression (Neuman & Baron, 1998), workplace bullying (Einarsen, 1999), incivility (Cortina et al., 2001), abusive supervision (Tepper, 2000), and sexual harassment (Sheffey & Tindale, 1992). While studies in these research streams provide valuable in-depth insights into each form separately, they also reveal common grounds among these types: all refer to negative social acts that detrimentally affect the well-being and performance of their targets, as well as the teams and organizations where the mistreatment occurs (Bowling & Beehr, 2006; Schilpzand et al., 2016). Several scholars have argued that rigidly categorizing these types may hinder knowledge development, as the distinctions often blur for individuals experiencing real-world situations (Aquino & Thau, 2009; Dhanani & Bogart, 2025; Verschuren et al., 2021).

There is substantial theoretical and operational overlap across subtypes of IWM (Dhanani & Bogart, 2025; Hershcovis, 2011). Over a decade ago, Hershcovis (2011) warned against the proliferation of semantically and operationally similar constructs, questioning the added value of such divisions. Yet, little has changed since then. If anything, more mistreatment types have emerged as distinct literature sections or have grown exponentially and continue to be studied in isolation, limiting cross-fertilization and impeding the cumulative advancement of the IWM field. Given the conceptual overlap—such as shared antecedents, conceptual definitions, and similar outcomes—and sometimes similar measurement items in scales across mistreatment forms, examining co-workers’ responses to each form in separate streams hampers a comprehensive understanding of how and why employees respond to IWM (Dhanani & Bogart, 2025; Dhanani & LaPalme, 2019; Hershcovis, 2011; Verschuren et al., 2021). This issue becomes even more pressing considering that co-workers typically do not draw the fine-grained distinctions that scholars do. This aligns with event-based models of intervention, which suggest that co-workers respond primarily to the perceived meaning of an incident rather than its formal classification (Bowes-Sperry & O’Leary-Kelly, 2005). This is further supported when taking a social information processing perspective, stating that “individuals, as adaptive organisms, adapt attitudes, behavior, and beliefs to their social context and to the reality of their own past and present behavior and situation” (Salancik & Pfeffer, 1978, p. 226). Because individuals adapt their attitudes and behaviors to their broader social context and personal experiences, co-workers witnessing IWM are likely to respond based on the perceived meaning and dynamics of the situation rather than the specific subtype of mistreatment (e.g., incivility vs. harassment). This supports the added value of studying co-worker reactions across all forms of IWM collectively, as it reflects how such events are interpreted and acted upon in real-world settings, i.e., through a systemic lens, rather than strict categorical distinctions.

To date, research on IWM has predominantly focused on its antecedents and consequences, underscoring the importance of preventing IWM and halting its progression when it occurs despite preventive efforts. Many antecedents of IWM have been identified within the organizational context (e.g., organizational change, leadership styles) and are related work stressors (e.g., role conflict, workload) (Balducci et al., 2021). However, IWM must be understood as a social phenomenon including various social actors, and shaped by broader organizational norms, group dynamics, and social structures, rather than being confined to isolated interactions between a perpetrator and a target (Hershcovis et al., 2020). Workplace culture, power hierarchies, and bystander behaviors all influence the emergence, maintenance, and mitigation of mistreatment, highlighting the collective and systemic nature of the issue (Vranjes et al., 2023a). This perspective has further inspired research into disentangling the role of co-workers witnessing IWM in its development and continuation.

The literature on IWM has applied various terms referring to co-workers that are aware of IWM, with a selection including bystanders (Gloor et al., 2023), observers (Reich et al., 2021), third parties (Dhanani & LaPalme, 2019), and witnesses (Báez-León et al., 2016). While labels like ‘bystanders’ might imply passivity, it is important to recognize that co-workers are not always mere spectators. They can actively engage in the situation, either by intervening to stop the negative interactions between the actor and the target or by exacerbating the situation (Banyard, 2011; McDonald & Flood, 2012; Reich & Hershcovis, 2015). The well-established framework of Paull et al. (2012) categorizes such co-worker behaviors into four quadrants. The first quadrant concerns the active-constructive roles. These co-workers take proactive steps to stop IWM, support the target, or report the mistreatment. They actively challenge harmful behavior and seek to foster a positive work environment. The second quadrant encompasses active-destructive roles: these individuals actively contribute to IWM by encouraging, assisting, or joining in the mistreatment. Their actions escalate the situation and reinforce the perpetrator’s behavior. On the passive side of the framework, the passive-constructive roles are those of people that do not intervene directly, but may privately sympathize with the target, offer support in non-visible ways, or disapprove of the IWM without taking action. Lastly, passive-destructive co-worker roles are assigned to those that ignore IWM, remain neutral, or choose not to acknowledge it. They are coined as ‘destructive’, as their inaction can contribute to the perpetuation of workplace mistreatment by signaling tacit acceptance. Thus, given that co-workers play a crucial influencing role in the evolution of the mistreatment process, they constitute a relevant actor in this social context (Andersson et al., 2009). For reasons of clarity in the variously applied terms, in this paper, we define the third party in the social situation of IWM simply as a ‘co-worker’. This term refers to any individual, regardless of their position, who receives information—either directly or indirectly—about an act of mistreatment towards a colleague, and who is neither the (initial) actor nor the target of the act.

Our paper reviews the current literature with a specific focus on how co-workers decide to react to their initial confrontation with IWM. Our aims are twofold: first, we seek to integrate the knowledge derived from the separate literature streams on the factors determining co-worker behaviors. Second, we aim to apply a decision-making lens, as there is limited focus on the specific processes co-workers undergo in their decision-making and how these processes manifest as a whole. This approach will enable us to create an integrative framework that can be utilized for the further development of co-worker intervention programs and the research field in general. This knowledge can then be applied in organizations, for example, through the development of co-worker intervention strategies. We thus conclude this paper with recommendations for both future research and practice.

## 3. Method

An integrative review was considered to be suitable for our aim: combining insights from diverse research streams by synthesizing the existing knowledge into a coherent conceptual framework (Snyder, 2019). We followed the guidance of Cronin and George (2023) on the purpose and methodology of integrative reviews and adopted the strategy of redirection, that is, uncovering new insights into co-worker reactions to IWM by gathering and integrating findings from fragmented literature streams. As a first step, we systematically identified and screened the literature based on predefined inclusion criteria (Torraco, 2005). In the next phase, we synthesized the disparate findings into an integrative framework that captured the decision-making processes underlying co-workers’ behavioral responses to IWM.

The studies forming the basis of our comprehensive review were identified through a systematic electronic search in Web of Science. We used the following keywords to guide our search: bystander(s); observer(s); witness(es); co-worker(s); third party(parties); spectator(s); outsider(s); accomplice(s); onlooker(s); audience. These keywords were combined with different types of IWM (‘AND’ function): bullying; incivility; harassment; counterproductive behavio(u)r; social undermining; cyberbullying; cyberabuse; cyberharassment; abusive supervision; interpersonal conflict; interpersonal deviance; injustice; violence; wrongdoing; abuse; aggression; mobbing; antisocial behavio(u)r; ostracism; sabotage; exclusion; rudeness; and mistreatment. The keywords were chosen in order to address the broad literature and involve the different subtypes of IWM, aiming to bridge the separation between the different research streams. Lastly, as this review aimed to specifically focus on interpersonal mistreatment within the work environment, the following keywords were also added (‘AND’ function): work; workplace; organi(s/z)ation(s/al).

The initial literature search included articles published up to July 2024. An initial update was conducted in December 2024. A final update of the literature search was performed during the review process, encompassing articles published up to April 2025. A total of 3602 records were found. After removing duplicates, 3570 articles remained. First, the titles and abstracts were screened for relevance. We assessed whether the publication concerned peer-reviewed, empirical research on the topic of co-worker reactions regarding the broad range of forms of IWM. For example, studies focusing on victims’ own reactions, mistreatment occurring outside the workplace (e.g., in schools or leisure settings), or cases involving perpetrators who are not part of the workforce (e.g., patients or clients) were excluded from further consideration. Second, we fully screened the papers that were considered for further analysis (N = 278). We focused specifically on factors influencing co-workers’ behavioral reactions and whether the type of reaction studied was sufficiently specific. Rather than examining general statements such as “co-worker would react”, we included studies that identified concrete behavioral reactions, such as support for the target or perpetrator, or inaction. Studies employing quantitative, qualitative, or mixed-method approaches were all considered. Finally, a total of 64 articles, including 85 research studies, were selected for this review. These 64 articles are marked in the reference list with an asterisk.

The selected articles were thoroughly reviewed, and all factors identified as relevant to co-workers’ decision-making processes were systematically extracted. Articles were included if they clearly indicated the type of co-worker response studied. Based on the co-worker responses examined within the retrieved articles, four categories were used to structure the data: target-supportive behaviors, retaliation against the perpetrator, perpetrator-supportive behaviors, and inaction. Additionally, two primary approaches to studying co-workers’ reactions became apparent: those examining actual behaviors (e.g., recall studies: what did a co-worker really do) and those focusing on intended responses (e.g., scenario-based studies: what would a co-worker do). Subsequently, all factors influencing the likelihood of certain behaviors were listed. These extracted factors were then categorized and specified per co-worker reaction category (target-supportive, perpetrator retaliation, perpetrator-supportive, no action) and whether the research focused on co-worker behavior or intention. Thematic analysis techniques were employed to conceptually structure the listed factors (Torraco, 2005). The results of the literature review were synthesized and a decision-making lens was applied in order to create a new, integrative way of looking at the topic of co-worker reactions to IWM (Torraco, 2005).

## 4. Results

In the first part of this section, we discuss the findings of the literature review. Our results yield eight categories regarding individual and contextual factors influencing co-workers’ initial responses related to individual (i.e., perceived responsibility, emotion and affect, and personal characteristics) and contextual factors (i.e., social relationships, risks and cost considerations, workplace characteristics, target characteristics, and perpetrator and IWM characteristics). Below, we elaborate on which factors have been studied in these categories and how they exert an influence on co-worker reactions, both as actual and intended behavior. In the second part of this section, we propose an integrative decision-making model for co-workers’ responses to initially witnessing IWM. This model incorporates additional, particularly relevant literature and provides a comprehensive synthesis of the identified factors in the literature review. It is important to note that the section of the results containing the integrative framework is not merely a presentation of findings from the literature review. Instead, it serves as a synthesis and discussion of these findings, adding a decision-making lens to better understand co-worker reactions. This approach allows us to move beyond a simple categorization of factors and offers a deeper understanding of the decision-making processes involved. The integrative framework thus provides a cohesive narrative that bridges the gap between the results and discussion section.

### 4.1. Individual Factors

#### 4.1.1. Perceived Responsibility

This category pertains to co-workers’ attributions and perceptions of responsibility and deservingness regarding instances of IWM, as well as their views on the roles of the target and the perpetrator. When targets are perceived as responsible for their mistreatment or as deserving of it, co-workers’ intentions lean more toward inaction (Pierce et al., 2004), or they may even engage in behaviors that support the perpetrator (both in intention (Mulder et al., 2016), and behavior (Zhou et al., 2021)). In cases where co-workers attribute a high degree of responsibility or deservingness to the target, they are less likely to support them, both in their expressed intentions (Hellemans et al., 2017; Mulder et al., 2008, 2016, 2017; Pierce et al., 2004) and in their actual behavior (Mitchell et al., 2015; Østvik & Rudmin, 2001; Zhou et al., 2021), or to retaliate against the perpetrator (Pierce et al., 2004). Also, when they hold external attributions towards the behavior of the perpetrator, they are less likely to engage in retaliatory intentions (Fiori et al., 2016). Conversely, when co-workers attribute responsibility for the mistreatment to the perpetrator (S. C. Chen & Liu, 2019) or argue that the target is undeserving of mistreatment (Mitchell et al., 2015), they are more likely to engage in retaliatory behaviors against them. Finally, a distinct dimension of responsibility concerns the expectation that targets themselves should take action to stop the mistreatment. When co-workers believe that it is the target’s responsibility to address and halt IWM, they are more likely to refrain from intervening (Thompson et al., 2020).

#### 4.1.2. Emotion and Affect

Regarding behaviors, emotional responses such as anger (A. Chen et al., 2024; Liang & Park, 2025; Mitchell et al., 2015; Zhang et al., 2020; deontic anger: Priesemuth & Schminke, 2019), negative emotions (Ghumman et al., 2016), and empathetic emotions towards the target (C. Chen et al., 2021; Liang & Park, 2025) increase target-supportive behaviors, as they may heighten moral concern and motivate intervention. Similarly, anger (Gabriel et al., 2024; Mulder et al., 2008, 2016, 2017; moral anger: Hall & Dhanani, 2023), negative emotions (Ghumman et al., 2016), experiencing tension (Báez-León et al., 2016), empathetic emotions (C. Chen et al., 2021; M. Weber et al., 2019; Wei et al., 2023), pity for the target (Mulder et al., 2008), and sympathy for the target (Mulder et al., 2016, 2017) influence the intention to support the target. Conversely, schadenfreude (a pleasant feeling stemming from others’ misfortunes; C. Chen et al., 2021), discomfort with conflict (Haynes-Baratz et al., 2022), associated stress with reporting (Kondziolka & Liau, 2021), and dispositional envy (Haq et al., 2024) in co-worker behavior and fear in both co-worker behavior (Y. Yu et al., 2022) as co-worker intention (Mulder et al., 2008, 2016) and schadenfreude (C. Chen et al., 2021) in co-worker intention reduce the likelihood of engaging in target-supportive responses. Experiencing anger (Mitchell et al., 2015; Zhang et al., 2020; moral anger: O’Reilly et al., 2016; Zhao et al., 2025; moral outrage (i.e., anger provoked by violation of a moral standard, Batson et al., 2007; Kay et al., 2023; Lin & Loi, 2021)) leads to retaliatory behaviors against the perpetrator, reflecting an ethical duty to address wrongdoing and seek to restore justice. Moral outrage (Lin & Loi, 2021) and moral anger (Zhao et al., 2025) strengthen the intention for perpetrator-retaliatory responses. In contrast, contentment (Mitchell et al., 2015) and dispositional envy and schadenfreude (Haq et al., 2024) are linked to perpetrator-supportive behaviors, reflecting satisfaction with existing power dynamics or resentment toward the target. The intention to support the perpetrator is similarly influenced by a lack of sympathy for the target, anger evoked by perceived target responsibility, and fear, suggesting that some co-workers may side with the perpetrator (Mulder et al., 2016). Finally, workplace anxiety (Huang et al., 2019) and fear (Zhang et al., 2020) drive inaction, as they lead to avoidance behaviors and prevent co-workers from intervening.

#### 4.1.3. Personal Characteristics

Demographic characteristics that are linked to the enactment of target-supportive behaviors are gender (with women being more likely to engage in supportive actions than men; Ghumman et al., 2024; Kondziolka & Liau, 2021), age (with older people being more likely; H. H. Yu, 2023), tenure (with those with longer tenure being more likely; H. H. Yu, 2023), grade level (with higher grade level more likely; H. H. Yu, 2023), and race (positive effect of being a member of a minority group in the context of racism; H. H. Yu, 2023). This is similar for target-supportive intentions regarding gender (Desrumaux et al., 2018; Hellemans et al., 2017; Mulder et al., 2017; Ryan & Wessel, 2012; Sinclair, 2021), age (Ryan & Wessel, 2012), tenure (Hellemans et al., 2017), and race (Rosette et al., 2013). Next, identity-related factors such as moral identity (Mitchell et al., 2015), feminist identity in the context of discrimination (Gloor et al., 2023), and sharing ethnic minority identity in the context of racism (Fernando & Kenny, 2023) positively contribute to target-supportive behaviors. Also, prosocial orientation (Ghumman et al., 2016), self-efficacy (Ghumman et al., 2016), ethical efficacy (Priesemuth & Schminke, 2019), expectancy of successful intervention (Liang & Park, 2025), personal sense of fairness (Haynes-Baratz et al., 2022), having a supervisory position (Østvik & Rudmin, 2001; H. H. Yu, 2023), perceived power to act (Ghumman et al., 2024; Haynes-Baratz et al., 2022; Hershcovis et al., 2017; Jönsson & Muhonen, 2022), religious commitment and shared religion (Ghumman et al., 2016), and the co-worker having served in the army (H. H. Yu, 2023) positively increase the likelihood of target-supportive behavior. Regarding target-supportive intentions, we find similar effects for moral identity (Jensen & Raver, 2021), self-efficacy (Gligor et al., 2023; Hellemans et al., 2017), perceived power to act (Hershcovis et al., 2017), and religious commitment (Ghumman et al., 2016). Additionally, reflections on one’s own motivation and recalling of mistreatment in which they did not act (Diekmann et al., 2013) increase target-supportive intentions, whereas these are decreased when traditionality is high (i.e., the acceptance of the traditional social practices of where one lives (Schwartz, 1992; Wei et al., 2023). High moral identity has been identified as a factor contributing to less perpetrator-supportive and perpetrator-retaliation behavior (Mitchell et al., 2015) as well as more perpetrator-retaliation behavior (Lin & Loi, 2021; O’Reilly et al., 2016) and intention (Jensen & Raver, 2021; Lin & Loi, 2021; Skarlicki & Rupp, 2010). Co-workers with rule-based moral thinking orientation are less likely to hold intentions to retaliate against the perpetrator (Lin & Loi, 2021). Lastly, rational cognitive processing (Skarlicki & Rupp, 2010) and traditionality (Wei et al., 2023) decrease perpetrator-retaliatory intentions. Belief in a just world (Zhou et al., 2021) increases perpetrator-supportive behaviors. Low self-esteem (Aftab et al., 2024), low core self-evaluation (Huang et al., 2019), and feeling powerless (Paull et al., 2020) lead to co-worker inaction, while race (whites in the context of racial slurs), low racial identification with the target, and high social dominance orientation (i.e., preference for sustenance of inequality between themselves and subordinate groups (Sidanius & Pratto, 1999)) contribute to intentions of remaining inactive (Rosette et al., 2013).

### 4.2. Contextual Factors

#### 4.2.1. Social Relationships

Several relational factors positively influence target-supportive behavior, including a good (Ghumman et al., 2016; Ryan & Wessel, 2012) or trusting relationship with the target (Haynes-Baratz et al., 2022) or workplace friendship with the target (D’Cruz & Noronha, 2011; Madden & Loh, 2020). A good relationship with the perpetrator negatively impacts target-supportive behaviors (Ghumman et al., 2016). However, a respectful relationship with the perpetrator can also increase target-supportive behavior by facilitating respectful conversations with the perpetrator about their behavior (Haynes-Baratz et al., 2022). The presence of others (Ryan & Wessel, 2012) negatively impacts target-supportive behavior, whereas the voice of others (Lutgen-Sandvik, 2006) has a positive impact; however, in the work by Báez-León et al. (2016), the voice of others only shows a positive impact on target-supportive intentions and not on actual behavior. Friendship with the target (Coyne et al., 2019; Priesemuth, 2013), and gender compositions—namely, both a male and female perpetrator mistreating a female target (Jensen & Raver, 2021), and a female co-worker witnessing mistreatment of a female target (Mulder et al., 2017)—are associated with stronger intentions to support the target. In the context of a supervisor as a perpetrator, intentions to help are higher when the perpetrator’s ethnicity is white and the target’s ethnicity is black (Gligor et al., 2023). Regarding perpetrator-retaliatory behavior, a high-quality leader–member exchange (LMX) diminishes the likelihood of retaliation (S. C. Chen & Liu, 2019). Identification with the perpetrator (Umphress et al., 2013) and perspective-taking of the perpetrator (Fiori et al., 2016) decrease intentions to retaliate against them, while identification with the target (Umphress et al., 2013) and hostility toward the perpetrator (Wei et al., 2023) increase these intentions. High LMX in one study leads to more inaction, both in the sense of behavior as intention (Azeem et al., 2024) and more perpetrator-supportive intentions and behavior in another (Liu et al., 2022). Lastly, the perceived outsider status of the target predicts more perpetrator-supportive intentions and behavior (Liu et al., 2022).

#### 4.2.2. Risks and Cost Considerations

Target-supportive behaviors are discouraged by the perceived costs of intervening (i.e., negative consequences such as others’ perceptions about them; Ghumman et al., 2016) and fear of retaliation (Báez-León et al., 2016; Haynes-Baratz et al., 2022; Jönsson & Muhonen, 2022; Kondziolka & Liau, 2021; Y. Yu et al., 2022), but also concerns about jeopardizing collegial relationships, wanting to avoid disempowering targets, and worrying about making things worse (Haynes-Baratz et al., 2022). Co-workers may also refrain from intervening if they assume that taking action will not help and that they lack skills for responding (Haynes-Baratz et al., 2022), or they perceive intervening as too difficult (Østvik & Rudmin, 2001). These considerations suggest that perceived personal or social risks can outweigh moral or prosocial motivations, contributing to inaction. Fear of retaliation from the perpetrator or organization (Paull et al., 2020), concerns about repercussions (Thompson et al., 2020), and expectations of negative career consequences and of social isolation (MacCurtain et al., 2018) increase the likelihood that co-workers will remain passive bystanders. Such concerns reflect a workplace climate where intervention is perceived as personally or professionally hazardous, potentially molding a culture of silence around mistreatment. Moreover, fear of retaliation (Báez-León et al., 2016) and stigma by association (Mulder et al., 2016) negatively influence target-supportive intentions, as co-workers may fear that aligning with the target will damage their own reputation or social standing. Anticipated stigma by association can also positively influence intentions to support the perpetrator (Mulder et al., 2016), suggesting that some co-workers may align with the perpetrator to protect their own social status or avoid marginalization.

#### 4.2.3. Workplace Characteristics

Numerous studies highlight the influence of social norms and workplace culture on co-workers’ behavioral and intentional responses to IWM. A respectful climate, a culture of listening, clear norms about appropriate behavior (Haynes-Baratz et al., 2022), a perception of overall fairness (Priesemuth & Schminke, 2019), perceived psychological safety at the organizational and collegial level, perceived supervisory safety and support (MacCurtain et al., 2018), and low perceived goal competitiveness (i.e., co-workers perceive that there are incompatible goals and rewards between the targets and themselves; C. Chen et al., 2021) positively impact target-supportive behavior by fostering an environment where intervention is expected and socially reinforced. Conversely, a workplace climate that tolerates IWM (Haynes-Baratz et al., 2022; Jönsson & Muhonen, 2022; Liang & Park, 2025) discourages support, normalizes mistreatment, and reduces co-workers’ willingness to support targets. Organizational identification (Gloor et al., 2023) and a lack of diversity among colleagues (Haynes-Baratz et al., 2022) decrease the likelihood of target-supportive behavior. Workplace characteristics also shape target-supportive intentions. An inclusive organizational culture (Collazo & Kmec, 2019), high workgroup identification (Báez-León et al., 2016), formal policies addressing mistreatment (Jacobson & Eaton, 2018), and low perceived goal competitiveness (C. Chen et al., 2021) are linked to stronger intentions to support targets, indicating that both explicit and implicit organizational signals foster prosocial responses. However, a high workload negatively impacts both target-supportive intentions and intentions to retaliate against perpetrators (Jensen & Raver, 2021), suggesting that significant job demands deplete employees’ cognitive and emotional resources for intervention. Regarding inaction, employees are more likely to refrain from responding to IWM when they perceive a lack of organizational support or low supervisory safety (MacCurtain et al., 2018).

#### 4.2.4. Target Characteristics

The target’s characteristics and the co-worker’s perception of them shape the co-worker’s response. The target’s ethnicity as well as their perceived foreignness reduce target-supportive behavior when the target is considered to be more foreign (A. Chen et al., 2024). High disruptiveness (i.e., the perception of the target’s stigmatized attributes as hampering or increasing the difficulty of having a relationship with the stigmatized person (E. E. Jones et al., 1984)) also negatively impacts the occurrence of target-supportive behaviors. Similarly, co-workers do not intend to support targets when they show low self-reliance (Mulder et al., 2017), show anti-social behavior (Desrumaux et al., 2016, 2018), and when the target is male (rather than female; Sinclair, 2021; M. Weber et al., 2019). Helping intentions increase when the target is revictimized (Desrumaux et al., 2016, 2018), when they appeal for help (Jensen & Raver, 2021), appear upset (Bowes-Sperry & Powell, 1999), engage in approach coping in the context of their mistreatment (Rae & Neall, 2022), and score well in their job performance (Jensen & Raver, 2021). Lastly, contrasting findings occur regarding the target’s sexual orientation: intentions to help are found to be decreased when the target is a lesbian (Meglich et al., 2020), whereas another study reports increased helping intentions when the target’s non-heterosexual orientation is known (Ryan & Wessel, 2012). The intentions to retaliate against the perpetrator increase when the target appeals for help (Jensen & Raver, 2021) and scores well in their job performance (Jensen & Raver, 2021; Umphress et al., 2013). Finally, perpetrator-supportive intentions are influenced by the target’s passive reaction (Diekmann et al., 2013).

#### 4.2.5. Perpetrator and IWM Characteristics

The final category encompasses characteristics related to the perpetrator and the mistreatment itself. Target-supportive behavior is most likely to occur when co-workers have experienced similar mistreatment themselves (Lutgen-Sandvik, 2006), have high recurrence beliefs (Ryan & Wessel, 2012), and when the perpetrator’s power is considered high (Jungert & Holm, 2022). Perceptions of the act of mistreatment also increase helping behaviors, namely, when the co-worker perceives the act to be discrimination (Gloor et al., 2023), when the intent to harm (Ghumman et al., 2024; Ryan & Wessel, 2012) and severity of harm (Ghumman et al., 2016) are high, and when ambiguity of the act is low (Ghumman et al., 2016). Similarly, target-supportive intentions increase when the co-worker perceives stronger moral violation (Hall & Dhanani, 2023) or the behavior to be an ethical issue (Bowes-Sperry & Powell, 1999), when there is low ambiguity of intent (Ghumman et al., 2016), high perception of harm (Jensen & Raver, 2021) or high perceived severity (Hellemans et al., 2017; Jacobson & Eaton, 2018), high recurrence beliefs (Ghumman et al., 2016; Ryan & Wessel, 2012), when the co-workers are victimized themselves (Feng & Savani, 2023; Gligor et al., 2023), and when a perpetrator makes an effort to rectify their behavior (Rae & Neall, 2022). The type of mistreatment also becomes relevant here: co-workers hold more intentions to support targets when the target explicitly attributes the mistreatment to discrimination (Sinclair, 2021), when co-workers perceive the act as racism (Gligor et al., 2023), when the origin of the mistreatment is person-related (rather than work-related (Coyne et al., 2019)), is expressed directly (rather than indirectly; (Ryan & Wessel, 2012)), is verbal (rather than exclusionary behaviors; (Ghumman et al., 2016)), when it concerns acts of aggression (rather than isolation; Desrumaux et al., 2016, 2018), or when it happens offline (rather than online; Coyne et al., 2019). Co-workers are more likely to retaliate against the perpetrator when acts of selective incivility are repetitive and personalized (Fernando & Kenny, 2023), the co-worker also becomes victimized (Zhang et al., 2020), and when the perpetrator’s power is high (Jungert & Holm, 2022); however, the findings of Y. Yu et al. (2022) show less retaliation when perpetrator power is high in the cases where co-workers share the same supervisor with the target. The co-worker’s intention to retaliate against the perpetrator increases with high perception of harm (Jensen & Raver, 2021) and perpetrator intent, especially when the target does not recognize the intended act as unjust (Umphress et al., 2013). Finally, and in contrast to the findings of Jungert and Holm (2022), MacCurtain et al. (2018) find that a perception of high perpetrator power increases the likelihood of inaction.

### 4.3. Towards an Integrative Framework

Our review reveals that most publications to date have focused on co-workers as active agents in halting IWM by supporting the target or retaliating against the perpetrator. The majority of studies have examined intention (N = 47; 56%) rather than actual behavior (N = 38; 44%). More importantly, only eight articles examined both co-worker intentions and actual behaviors concurrently. This is striking given the intention–behavior gap: factors influencing co-workers’ intentions do not necessarily predict actual behavior (Tenbrunsel et al., 2010). For example, a meta-analysis finds that fear of retaliation predicts the intention to report organizational wrongdoing but not the actual behavior (Mesmer-Magnus & Viswesvaran, 2005). This underscores the risk of examining intentions in isolation and basing intervention programs solely on intention-related factors (e.g., co-worker workload, schadenfreude, revictimization), which may not effectively promote active responses from co-workers (Sheeran & Webb, 2016). It is therefore essential to disentangle the factors influencing intention, behavior, and the transition between the two from the co-worker’s perspective.

To maintain conciseness, we focus on reporting significant findings; yet, the review also reveals some inconsistencies across studies. First, some factors are not consistently found to be significant across different studies. Second, certain factors yield mixed or contradictory results, indicating a lack of consensus on their role in influencing co-worker behavior. For example, high perpetrator power has been associated with both increased (Jungert & Holm, 2022) and decreased (Y. Yu et al., 2022) retaliation against a perpetrator, as well as increased inaction (MacCurtain et al., 2018). Similarly, the target’s sexual orientation has been found to both increase (Ryan & Wessel, 2012) and decrease (Meglich et al., 2020) intentions to offer support when non-heterosexuality is disclosed. These discrepancies suggest the presence of underlying moderators or inhibitors that shape co-worker reactions to IWM. They underscore the need for a more systematic and integrative approach, as studying the factors in isolation risks overlooking the complex interplay of elements influencing co-worker behavior. Therefore, it is essential to examine co-workers’ decision-making processes more comprehensively, taking into account the interplay of relevant factors. Such an approach offers a clearer insight into what shapes co-worker reactions in the context of IWM. Additionally, integrating a decision-making lens into our analysis allows us to move beyond mere description and towards a more explanatory and predictive model. This model can account for the variability in co-worker responses by incorporating cognitive and affective processes, thereby offering a more nuanced and actionable framework for both researchers and practitioners. Advancing our literature review findings, we add a decision-making lens and propose a framework that distinguishes between intentions to intervene and actual behavior, acknowledging their distinct roles in the IWM process and how they should be considered in future research and practical applications.

#### 4.3.1. From Intention to Response: Dual Processing Accounts

**Figure 1:**
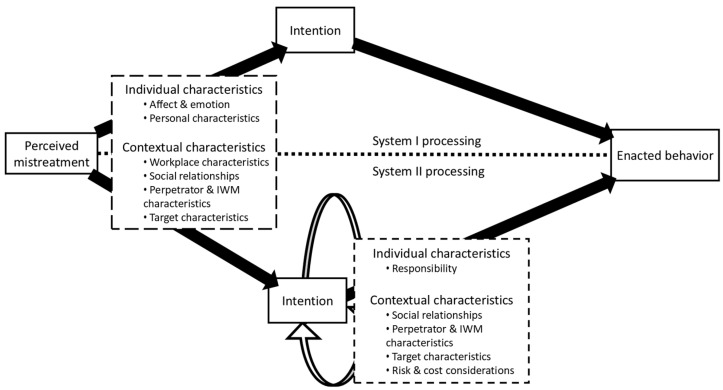
An integrative framework approaching factors influencing co-workers’ decision-making processes from a dual processing lens.

A particularly suitable avenue to thoroughly understand the differences between intention and behavior, and their linkage, lies in dual processing accounts of human behavior (Stanovich, 1999). This approach, especially when separating intention and actual behavior, sheds light on immediate/automatic versus deliberate/conscious actions (Evans & Stanovich, 2013). ‘System I processing’ is fast, unconscious, automatic, and primarily linked to emotional responses (Evans, 2008), leading co-workers to immediately decide on a behavioral response when witnessing IWM. In contrast, ‘system II processing’ is slow, deliberate, and requires more cognitive effort (Evans, 2008), allowing co-workers to gather additional information and engage in a conscious, cognitive process before deciding how to respond to the witnessed IWM. Despite its obvious relevance, the dual processing perspective on co-workers’ decision-making in IWM contexts has received rather limited attention (Dhanani & LaPalme, 2019; Skarlicki & Rupp, 2010). Expectations are mixed: Dhanani and LaPalme (2019) suggest that co-worker interventions mostly stem from a system II process, while system I processes fuel moral judgements and emotions. Conversely, Skarlicki and Rupp (2010) have found higher retribution tendencies toward perpetrators when system I processing is primed. Our framework incorporates both system I and system II processing in the context of co-worker behavior, as illustrated in Figure 1.

#### 4.3.2. System I Processing

The first part of our framework (see the top part of Figure 1) is associated with the decision on intention and the linear continuation of enacted behavior, often seen as intuition (Evans, 2010). The brain dictates immediate actions without the need or desire for additional information, effectuating the intention rapidly and autonomously (Skarlicki & Rupp, 2010). This implicit and automatic processing is linked to evolutionary responses and emotions (Evans, 2008). Emotions provide immediate, intuitive responses to social situations (Bastick, 1982), which can either facilitate or hinder co-worker intervention. Affective states significantly influence whether co-workers perceive mistreatment as requiring intervention or if they distance themselves from the situation (Maitlis et al., 2013). This can be understood along the lines of affective events theory (AET; Weiss & Cropanzano, 1996), positing that workplace events—such as witnessing mistreatment—trigger affective reactions, which in turn shape employees’ attitudes and behaviors. By emphasizing the situational triggers of affect, AET explains why different co-workers react differently to the same mistreatment event (Maas & van den Bos, 2009): some with empathy and intervention, others with fear and withdrawal. Thus, AET underscores the role of emotions in shaping workplace interactions and explains why responses to mistreatment are often immediate and driven by affective, rather than rational, deliberation.

AET suggests that workplace experiences generate emotions based on individuals’ subjective interpretations, which can fuel affect-driven intuitive responses (Weiss & Cropanzano, 1996). This becomes clear in the results of this review where emotions such as anger, sympathy, and empathy for the target increase active behavior that is oriented towards restoring justice, whereas feelings of fear, discomfort, and stress lead to avoidance rather than target support. Unexpected and socially salient workplace mistreatment may trigger stronger affective reactions than expected mistreatment or when IWM is the norm (van Kleef et al., 2015). In system I processing, co-workers heavily rely on the social context (Evans, 2003), especially if it contrasts with their expectations. This clarifies why psychologically safe climates in an organization with clear behavioral norms increase the chances of target-helping behavior, and those where co-workers lack the perception of support are more related to inaction. The characteristics of mistreatment also play a crucial role in determining the difference between baseline norms and the incident. The severity and intentionality of the acts increase the perception of injustice and thereby may increase the experienced negative effect and the tendency to restore justice (Weiss et al., 1999). Additionally, the closeness of co-workers to the involved actors strongly influences their emotional response. Humans are more affected by injustice toward those they identify with or are close to (Tone & Tully, 2014). This implies that the category of social relationships significantly influences co-workers’ intuitive responses to IWM: close relationships or identification with the target fuels target-supportive or perpetrator-retaliatory behaviors, while closeness to the perpetrator more likely leads to perpetrator-supportive behavior or inaction. Personality characteristics, especially those related to justice motives and identification with the actor or target groups, are also highly relevant in this decision-making process, as they also fuel the experienced states of affect when witnessing acts of IWM. Those more sensitive to injustices are more likely to intuitively react to restore justice (target-supportive or perpetrator-retaliatory) (Barclay et al., 2005). System I processing is thus mainly characterized by an affect-based decision-making approach (E. U. Weber et al., 2004).

#### 4.3.3. System II Processing

The second part of our framework (see the bottom part of Figure 1) is associated with a more cognitive, judgmental decision-making process, characterized by extended time and a prolonged analysis of information (Stanovich, 1999). The response is not immediate or automatic; instead, it involves co-workers gathering and considering additional information. While an intention may form in the moment, this does not guarantee immediate intervention (Sheeran & Webb, 2016). Co-workers are not required to act during the mistreatment itself; they continue to interact with the work environment, potentially experiencing further mistreatment and engaging with the perpetrator, target, and other co-workers. As a result, decisions can evolve over time, with actions potentially occurring later, or not at all (Moghavvemi et al., 2015). The qualitative research that is part of this review shows this process more in depth. The recurrence of acts of IWM creates the opportunity for bystanders to change their intention (Lutgen-Sandvik & Fletcher, 2013). When co-workers decide not to act immediately, they often start to make attributions about the situation. Attribution theory (Heider, 1958) explains how people interpret and assign causes to others’ behaviors and experiences. In the context of IWM, co-workers evaluate whether the target’s suffering is due to internal factors (e.g., their personality, actions, or incompetence) or external factors (e.g., an unfair supervisor, organizational culture, or situational pressures). When bystanders attribute the mistreatment to internal factors, they may perceive the target as partially or fully responsible for their negative treatment (Hellemans et al., 2017). This internal attribution reduces empathy and makes bystanders less likely to intervene, as they may believe the mistreatment is a logical or justified consequence of the target’s behavior (Betancourt, 1990). Additionally, attributing mistreatment to internal factors allows co-workers to maintain a psychological distance from the situation, reducing their sense of responsibility to act (Kim & Kim, 2023). It also helps them avoid potential social risks, such as retaliation from the perpetrator or negative repercussions for siding with a stigmatized target (Mulder et al., 2016). As a result, target-blaming attributions serve as a justification for less target-supportive and perpetrator-retaliatory responses, and more perpetrator-supportive responses or inaction. Additionally, when targets do not actively resist or respond to mistreatment, co-workers may interpret this passivity as implicit acceptance of the behavior. This highlights the risk of bystanders attributing silence or inaction as consent. Thus, when co-workers perceive a target of IWM as deserving of, responsible for, or accepting of the mistreatment, their likelihood of offering support decreases, and as a result, the likelihood of perpetrator-supportive behaviors increases.

Applying the results of our review, they clearly show the attributional effects of the perceived responsibility of the co-worker regarding the involved actors: if the targets are seen as responsible or deserving, they are less likely to receive help. Moreover, when the perpetrator is seen as responsible, they are likely to receive more retaliation. The characteristics of the perpetrator, the acts of IWM, and the target also play a role. Perpetrator characteristics, such as status or perceived intent, can influence whether co-workers see the mistreatment as an abuse of power or as justified behavior, influencing their attributions of the situation. Mistreatment characteristics, including severity, recurrence, and ambiguity, shape whether co-workers perceive the actions as deliberate harm or a misunderstanding, again adding to how and to whom they attribute responsibility. Lastly, target characteristics, such as job performance or self-reliance, affect whether bystanders attribute responsibility to the target or recognize them as unfairly targeted. Highlighting this point underscores the complexity and subjectivity of attributional processes, illustrating that co-worker responses are influenced not only by the act of mistreatment itself but also by the broader social and contextual factors (Rudolph et al., 2004). This perspective aligns with attribution theory, reinforcing that workplace mistreatment is interpreted through a lens shaped by multiple interacting variables. Taking these factors together, we can state that the decision-making approach here is not (solely) affect-based, as in system I processing, but is rather recognition-based (E. U. Weber et al., 2004). Incorporating these factors—such as the perceived responsibility of the target, the characteristics of the perpetrator, and the mistreatment itself—requires individuals to classify the situation into known categories, activating recognition-based decision rules. These classification processes align with E. U. Weber et al.’s (2004) recognition-based decision-making framework, where decisions are driven by previously learned patterns or social roles, rather than purely affective, intuitive responses. Thus, the decision-making process shifts from being primarily affect-driven (system I) to a more structured, rule-based approach that considers the context and the social roles involved.

Moreover, when one engages in system I processing, there is no time to think about the consequences of one’s behavior. However, when co-workers engage in system II processing, they take the time to consider their preferred response, but also the consequences of this response become more salient (Evans, 2008). This becomes evident in the factors found in this review related to risks and cost considerations. When these risks and costs are accounted for, co-workers show less target-supportive and perpetrator-retaliatory responses, and more inaction and perpetrator-supportive responses. Attribution theory explains that when co-workers attribute the perpetrator’s behavior to external situational factors, they may perceive intervention as unjustified or even counterproductive, increasing their hesitation due to the risk of social or professional repercussions (Heider, 1958). Conversely, if they attribute the mistreatment to internal factors (e.g., the perpetrator’s malicious intent), they may recognize a moral imperative to intervene; however, this recognition is still tempered by considerations of retaliation, organizational norms, and potential career consequences (Martinko et al., 2011). Thus, attribution theory helps explain why deeper, deliberate processing leads co-workers to weigh risks and costs more heavily: internal attributions may sustain moral outrage but still be overridden by pragmatic concerns, while external attributions may lead to the justification of inaction or even perpetrator-supportive behaviors (Rudolph et al., 2004). Here, decision-making stems more from a calculation-based origin, weighing the costs and benefits of the expected outcome of a decision, rather than an affect-based approach (E. U. Weber et al., 2004).

## 5. Discussion

### 5.1. General Discussion

We conducted an integrative review on co-worker reactions to witnessing IWM. By incorporating various literature streams and synthesizing the findings into an integrative framework, we are contributing to the essential cross-fertilization across research silos in the IWM literature (Dhanani & Bogart, 2025). Utilizing a decision-making lens—specifically dual processing accounts of human behavior—we elucidate the importance of distinguishing between factors influencing behavioral intention and those affecting actual behavior, and how co-workers make decisions when witnessing IWM (Evans, 2008; Sheeran & Webb, 2016). Affective events theory (Weiss & Cropanzano, 1996) and attribution theory (Heider, 1958) provide valuable insights into which factors identified in this review are most relevant when a co-worker engages in system I or system II processing. When applying dual processing accounts, it is crucial to note that co-workers may differ in their preferred—and thus, most frequently used—processing system (Evans, 2003). For instance, individuals with a high need for cognition have a strong desire to engage in and enjoy effortful cognitive activities, making them more likely to employ system II processing (Cacioppo & Petty, 1982). Conversely, those with high faith in intuition trust their gut feelings and tend to rely on immediate, automatic judgments characteristic of system I processing (Epstein et al., 1996).

For future research and the application of this framework in the development of co-worker intervention training, it is essential to recognize that responses can originate from either system I or system II processing. Consequently, altering decision-making processes can be more challenging for co-workers utilizing a system I approach compared to those employing a system II approach. Decisions made through system I processing are often guided by heuristics and emotional responses, making them rather deeply ingrained and more resistant to change (Evans, 2008). This resistance arises because intuitive judgments are typically based on personal experiences and affective reactions, which are less susceptible to external information or logical persuasion (Bastick, 1982). In contrast, when individuals engage in system II processing, they are more open to incorporating additional information, especially if it is presented logically and aligns with their existing knowledge structures (Evans, 2008). This openness facilitates the modification of decision-making processes, such as becoming more empathetic and supportive towards victims of IWM when provided with compelling facts and arguments (Evans & Stanovich, 2013).

To influence decision-making effectively, particularly in contexts like promoting support for victims of workplace mistreatment, it is crucial to consider the cognitive mode in which individuals are operating. Targeting system I and thereby changing intuitive judgments may require interventions that address emotional responses and personal biases. This could involve storytelling, emotional appeals, or experiential learning that resonate with individuals’ existing feelings and experiences. When targeting system II, influencing decisions through analytical reasoning involves presenting clear, logical, and evidence-based information. Providing data, structured arguments, and opportunities for critical thinking can facilitate the adoption of new perspectives and behaviors (Evans & Stanovich, 2013).

One could argue that decisions resulting from system II processing are more desirable in this context than those originating from system I processing, as the latter is typically characterized by affective, justice-driven decision-making (Dhanani & LaPalme, 2019). Specifically, co-workers who actively support the target are often motivated by a desire for retaliation against the perpetrator, which may lead to more antisocial responses and potentially contribute to a spiral of negative workplace behaviors (Vranjes et al., 2023b). Decisions rooted in system II processing, despite being subject to cognitive biases, might provide opportunities to offer alternative perspectives, interrupt toxic spirals, and contribute to the creation of a safer and more positive work environment where unwanted behavior and experiences can be discussed and constructively handled (Evans, 2008). However, two factors may limit the extent of active intervention from co-workers. First, the calculation-based approach of system II decision-making may result in less intervening, as co-workers might perceive the anticipated costs as outweighing the potential benefits (E. U. Weber et al., 2004). Additionally, as co-workers share the same workforce as both the target and the perpetrator, they may continuously receive new information, which can lead to information overload. This can result in paralysis by analysis, causing a state of indecision and an inability to choose a course of action (Newark, 2014).

### 5.2. A Future Research Agenda

An integrative review should stimulate further research on the topic (Torraco, 2005); therefore, we offer some clear recommendations and research questions for the future.

Several limitations must be considered when utilizing this framework for future research or applications. First, the framework begins when a co-worker directly or indirectly witnesses an act and interprets it as IWM, thereby bypassing the sensemaking process, where a co-worker decides whether to interpret an event as mistreatment. A first research avenue for the future is thus to study how and why co-workers come to interpret behavior as IWM. This is a research avenue that warrants further exploration, as perceptions can vary significantly across individuals and contexts (D. A. Jones & Skarlicki, 2013; Rotundo et al., 2001). Understanding when and why co-workers perceive and interpret such acts as mistreatment can inform strategies to enhance the recognition of these acts and raise awareness of the need for intervention (Bowes-Sperry & O’Leary-Kelly, 2005; O’Reilly & Aquino, 2011). We look at IWM as an umbrella term as this seems most fitting for research on co-workers, and thus we suggest further research on if and when co-workers perceive acts as IWM, for example, by building on the dimensions identified by Dhanani and Bogart (perpetrator motivation, perpetrator-target contact, magnitude of harm, and prohibition of the behavior; 2025).

Second, as our aim was to conduct an integrative review, a statistical analysis was not performed. However, throughout the process, we noticed that further statistical analysis would be difficult due to the scattering of concepts and measurements. Most studies in our review utilize ad-hoc measurement methods of co-worker reactions. Not all four types of co-worker responses are measured in every study, nor is there a standard for how these responses are measured, and full transparency of measurement items is not always achieved. This inconsistency makes it difficult to compare findings across different studies. This raises the question of how co-worker reactions can be measured reliably. We call for the development and use of validated measurements for co-worker reactions, to ensure that future research is more comparable and facilitates meta-analytical analyses.

Third, this framework provides insights into the decision-making process leading to an initial response from co-workers when witnessing IWM. Applying this knowledge in the development of co-worker intervention programs is particularly valuable, as it may help halt IWM early in the process and prevent further escalation. However, it is crucial to recognize that the involved parties often remain in the same workplace, and interactions continue beyond the initial response. Currently, little is known about this further process, leading to new research questions on how co-workers make sense of situations, after they decide on their own (in)action and IWM persists. Interventions should also support co-workers in how to intervene after they have already taken action or remained passive, even after IWM persists. Further research is needed to elaborate on the sensemaking process of co-workers following their initial response, and the long-term involvement of co-workers in cases of IWM (Ng et al., 2020).

Despite extensive research on co-worker reactions to IWM, our review reveals that not all categories of factors related to the four types of co-worker responses have received equal empirical attention. Most notably, the results indicate that target-supportive reactions, both intentional and behavioral, have garnered significantly more attention in current research practices than other types of reactions. This focus is understandable, as these are the behaviors we aim to encourage. However, to fully comprehend co-worker decision-making processes, it is crucial to also gather knowledge on the factors that shape other, potentially less desirable, co-worker responses. For instance, the current research remains unclear about which workplace characteristics may foster perpetrator-supportive intentions or behaviors. Consequently, interventions in organizations aimed at halting IWM may be less effective if the workplace characteristics that increase the likelihood of perpetrator-supportive behavior are not addressed (Banyard, 2024). Understanding why co-workers behave the way they do, across the spectrum of possible reactions, will provide the most reliable basis for developing successful co-worker interventions. Future research should thus focus on exploring when and why co-workers engage in different reactions than those supportive of a target. Given the current underrepresentation of perpetrator-supportive behaviors, we advocate for placing them at the forefront of future research agendas.

Regarding the different categories of factors, there is a strong focus on the personal characteristics of the co-worker. While this is analytically interesting, it offers limited value for developing co-worker intervention programs, as biological characteristics and personality traits are not easily altered (Harris et al., 2016). Given that IWM is a social phenomenon (Hershcovis et al., 2020), future research could significantly enhance our understanding of co-worker reactions by addressing influencing factors related to the social context and incorporating them into intervention programs. We pose that studying the role of the social and organizational context in the execution of co-worker interventions, as well as how these can be altered to stimulate prosocial intervention strategies, is crucial to further advance the field. For example, although target characteristics have been examined in relation to co-worker intentions to intervene, their influence on actual co-worker behavior remains largely unexplored. Investigating these dynamics could provide valuable insights into whether strong workplace relationships with the perpetrator discourage intervention, whether social bonds contribute to co-worker inaction, or whether certain relational factors might even encourage the justification or reinforcement of mistreatment. Understanding these social influences could inform the development of more effective intervention strategies that address the broader social context within which IWM occurs.

It is important to note that the majority of studies included in this review originate from Western countries, particularly North America and Western Europe. This can pose a significant limitation as the findings are thus embedded within specific cultural, organizational, and legal contexts that may not be universally applicable. Cultural norms can significantly shape how co-workers perceive and respond to mistreatment. As such, the current evidence base may not fully capture the diversity of co-worker responses across global contexts. Future research could thus engage in the role of cultural and regional differences and their influence on co-worker reactions to IWM, as well as the extent to which existing findings from Western contexts are generalizable across diverse cultural settings. To advance the field and enhance the generalizability of findings, future research should prioritize cross-cultural studies and include underrepresented regions to better understand how cultural values and workplace norms influence co-worker decision-making in the face of IWM.

Lastly, our review indicates that only a few studies specifically examine factors likely related to system II processing. These studies demonstrate that co-workers can be encouraged to think more deliberately about their decisions and thus engage in system II processing. This can be achieved, for example, by reflecting on their own motivation (Diekmann et al., 2013), stimulating rational processing rather than experiential processing (Skarlicki & Rupp, 2010), and even by triggering a recall of previous IWM acts in which they did not take action (Diekmann et al., 2013). This is unsurprising, as most research—particularly quantitative scenario-based studies—employs an event-based approach that does not necessarily allow co-workers to seek additional information to comprehend the situation. Therefore, we advise that future paths should explore the use of different research methods to answer the remaining questions regarding co-worker interventions when witnessing IWM. Qualitative research and longitudinal studies (such as event-based diary studies) can offer more insights into these mechanisms of system II processing.

### 5.3. Practical Recommendations

From a practitioner perspective, it is valuable to look at IWM as an umbrella concept, rather than focusing on and addressing the various subtypes separately. Current organizational policies and regulations address workplace mistreatment from a more holistic perspective, focusing on the general negative social environment rather than subtypes specifically (ILO, 2022). Data on employee experiences with IWM also show that exposure to toxic behaviors often involves more than just one type (SERV, 2023). Therefore, we underscore the need to integrate the different research silos when making recommendations for practice.

In professional practice, these recommendations can be applied by human resources (HR) professionals, occupational health services, and managers and employers to design and implement comprehensive IWM prevention and intervention programs. These strategies can be used to create a positive work environment and address IWM proactively. All employees can benefit from understanding their role in preventing and responding to IWM, fostering a more inclusive and respectful workplace.

To effectively address IWM, practitioners can implement several practical strategies based on the integrative framework presented in this paper. First, a supportive organizational culture is a critical step. Organizations should develop and communicate clear policies and procedures regarding IWM, including robust reporting mechanisms and defined consequences for perpetrators. Fostering a culture of psychological safety, where employees feel secure in reporting IWM without fear of retaliation, is crucial. Addressing fears of retaliation by ensuring confidentiality in reporting and providing protection for those who report IWM can mitigate risks and cost considerations. The leadership’s commitment to addressing IWM and modeling appropriate behavior sets a strong example for the entire organization.

Next, co-worker intervention training and awareness programs are essential. Educating employees about the dual processing model—distinguishing between system I (fast, automatic, emotion-driven responses) and system II (slow, deliberate, cognitive responses)—can help them understand their own decision-making processes. Role-playing scenarios and providing specific strategies for different situations can be particularly beneficial. Using the integrative framework in these role-playing exercises can help create awareness for co-workers on how different factors influence their reactions. This way, co-workers can experience the difference and become more aware of what supports or inhibits target-supportive behavior. In the case of inhibiting organizational factors, they then can be indicated and addressed, to create a supportive organizational culture in which co-worker interventions become more likely. Role-playing exercises have already been proven helpful in interventions for targets to recognize and address incivility (Lasater et al., 2015). Moreover, the personal development and empowerment of employees are also important. Encouraging employees to take ownership of creating a respectful workplace and empowering them to speak up against IWM can foster a proactive environment. Providing training on conflict resolution, communication skills, and assertiveness can help employees handle IWM situations more effectively. These initiatives should equip employees with the skills to intervene safely and effectively when they witness IWM.

Monitoring and evaluation are vital for continuous improvement and the evaluation of interventions (Nielsen et al., 2010). Regular surveys and feedback mechanisms can help assess the prevalence of IWM and the effectiveness of interventions. Implementing a system to track incidents of IWM and monitor the outcomes of reported cases ensures accountability and helps identify areas for improvement. Establishing support systems, such as peer support groups or designated IWM officers, can offer guidance and assistance to those affected by mistreatment.

While this review primarily focuses on co-worker interventions in response to the occurrence of IWM, the importance of prevention should not be overlooked. Preventive strategies aim to address the root causes and organizational conditions that give rise to mistreatment in the first place. By fostering inclusive cultures, promoting respectful communication, and implementing proactive policies, organizations can reduce the likelihood of IWM incidents altogether. Effective prevention not only minimizes harm but also reduces the burden on co-workers to intervene, thereby creating a healthier and more sustainable work environment.

## 6. Conclusions

We conducted a thorough integrative review of the current literature on co-worker responses to IWM and categorized the studied factors in eight categories. We found that there are differences between factors leading to co-worker intentions and those leading to actual co-working behaviors. Dual processing accounts shed light on why this is the case. For system I processing, we rely on affective events theory, and we explain how co-workers form a preliminary intention for a specific behavior upon witnessing mistreatment, i.e., an affect-based decision-making approach. Subsequently, they may follow a sequential, linear process and enact their decision without further consideration of additional information. Alternatively, relying on system II processing and attribution theory, we argue that co-workers may consider other factors and information before deciding whether to enact their initial intention or choose a different course of action, often relying on a recognition-based or calculation-based decision-making approach. This framework integrates current knowledge and addresses avenues for future research and practical implications, especially in the context of the development of co-worker interventions.

## Data Availability

The data supporting this study can be obtained from the corresponding author upon reasonable request.

## Abbreviations

The following abbreviations are used in this manuscript:

AET	Affective Events Theory
HR	Human Resources
ILO	International Labor Organization
IWM	Interpersonal Workplace Mistreatment
SERV	Sociaal-Economische Raad van Vlaanderen
